# EEG biomarkers analysis in different cognitive impairment after stroke: an exploration study

**DOI:** 10.3389/fneur.2024.1358167

**Published:** 2024-05-06

**Authors:** Mengxue Xu, Yucheng Zhang, Yue Zhang, Xisong Liu, Kunqiang Qing

**Affiliations:** ^1^Department of Neurology, Chongqing Public Healthy Medical Center, Chongqing, China; ^2^Department of Mathematics, College of Natural Sciences, University of Texas at Austin, Austin, TX, United States; ^3^Department of Psychology, School of Psychology, Shenzhen University, Shenzhen, China; ^4^Intensive Care Unit, Chongqing Public Healthy Medical Center, Chongqing, China; ^5^Automotive Software Innovation Center, Chongqing, China; ^6^Research Group of Brain-Computer Interface, Brainup Institute of Science and Technology, Chongqing, China

**Keywords:** stroke, cognitive impairment, EEG, PSD, functional connectivity

## Abstract

Stroke is a cerebrovascular illness that brings about the demise of brain tissue. It is the third most prevalent cause of mortality worldwide and a significant contributor to physical impairment. Generally, stroke is triggered by blood clots obstructing the brain’s blood vessels, or when these vessels rupture. And, the cognitive impairment’s evaluation and detection after stroke is crucial research issue and significant project. Thus, the objective of this work is to explore an potential neuroimage tool and find their EEG biomarkers to evaluate and detect four cognitive impairment levels after stroke. In this study, power density spectrum (PSD), functional connectivity map, and one-way ANOVA methods were proposed to analyze the EEG biomarker differences, and the number of patient participants were thirty-two human including eight healthy control, mild, moderate, severe cognitive impairment levels, respectively. Finally, healthy control has significant PSD differences compared to mid, moderate and server cognitive impairment groups. And, the theta and alpha bands of severe cognitive impairment groups have presented consistent superior PSD power at the right frontal cortex, and the theta and beta bands of mild, moderated cognitive impairment (MoCI) groups have shown significant similar superior PSD power tendency at the parietal cortex. The significant gamma PSD power difference has presented at the left-frontal cortex in the mild cognitive impairment (MCI) groups, and severe cognitive impairment (SeCI) group has shown the significant PSD power at the gamma band of parietal cortex. At the point of functional connectivity map, the SeCI group appears to have stronger functional connectivity compared to the other groups. In conclusion, EEG biomarkers can be applied to classify different cognitive impairment groups after stroke. These findings provide a new approach for early detection and diagnosis of cognitive impairment after stroke and also for the development of new treatment options.

## 1 Introduction

Although the mortality rate of stroke has declined dramatically with the development of stroke research and advances in therapeutic techniques (1–4), disability from stroke continues to plague those who recover from stroke, with studies suggesting that as many as 30% of stroke patients have disability after recovery (5). This disability includes both post-stroke physical disability and post-stroke cognitive impairment (PSCI). PSCI is defined as any severity of cognitive impairment, regardless of cause, noted after an overt stroke (6, 7). This cognitive impairment involves multiple cognitive domains, with executive dysfunction being the primary and core symptom of PSCI (8–10). Depending on the severity of the cognitive impairment, PSCI can be categorized as mild, moderate, and severe. Mild PSCI consists of mild cognitive impairment that does not yet meet the diagnostic criteria for dementia. This may be manifested as memory loss, inattention, etc.; Moderate PSCI stage has more severe cognitive impairment, which may involve multiple cognitive domains, such as memory, language, and judgment. At this stage, the patient’s daily life may be significantly affected. Cognitive function is severely impaired when reaching severe PSCI, and the diagnostic criteria for dementia have been met. Patients may experience severe memory loss, language impairment, and behavioral abnormalities (11–14). Multiple studies have consistently demonstrated that individuals diagnosed with PSCI exhibit poorer rehabilitation outcomes related to physical function. Furthermore, they have a reduced likelihood of resuming a normal social life and exhibit a significantly higher mortality rate compared to those who do not have PSCI (15–18). Therefore, timely and accurate assessment of PSCI is extremely important, as it will help in the prevention and intervention of PSCI.

Currently there are two main types of assessments of the PSCI. One is questionnaire-based neuropsychological assessment, such as the Mini-mental State Examination (MMSE) and scales such as the Montreal Cognitive Assessment Scale (MoCA). However, this type of assessment is highly subjective, and the accuracy and reliability of the assessment results are more questionable. In contrast, biomarker-based assessment may be more objective, accurate and reliable. Specifically, EEG uses low-resistance electrodes placed on the human scalp to record oscillations generated by potential changes in the brain (19). EEG is a widely used non-invasive method for cognitive neurological research due to its high temporal resolution, ease of use, and low cost. A study that developed quantitative EEG (QEEG) to characterize EEG waves in post-stroke patients at risk of developing vascular dementia found that compared to normal subjects, patients with post-stroke with mild cognitive impairment had higher delta relative power, while alpha and beta relative power was lower in patients with post-stroke with mild cognitive impairment compared to normal subjects (20, 21). The study also examined the relationship between brain regions. The study also examined coherence between brain regions, with patients with post-stroke with mild cognitive impairment exhibiting lower interhemispheric and intrahemispheric coherences. Furthermore, event-related potentials (ERPs) were found to be lower in patients with mild cognitive impairment, while the relative power of alpha and beta was higher (20). ERPs derived from EEG have also been used to assess cognitive function in stroke patients. The P300 is sensitive in detecting subtle PSCI and can be used as an important marker for assessing PSCI, while P3 latency is an important marker of recovery from cognitive dysfunction after stroke (22, 23). From the perspective of the fNIRS, the PSCI group had lower intra-right and interhemispheric functional connectivity (FC) than healthy controls (HC). In the PSCI group, specific brain areas, such as the somatosensory cortex and prefrontal cortex, had considerably lower FC (24). Interestingly, neither acute ischemic stroke (AIS) patients nor the HC group showed prefrontal cortex (PFC) activation throughout the test in another study by researchers. (25). Arenth et al. found substantial changes in deoxy-Hb levels between aphasic and non-aphasic groups, but no differences between HC and non-aphasic stroke patients (26, 27). fMRI is another non-invasive neuromonitoring technique for monitoring blood oxygenation. fNIRS and fMRI are closely related, and studies have shown a significant correlation between the hemodynamic response measured by fNIRS and the blood oxygen level-dependent (BOLD) response obtained by fMRI (28). One resting-state fMRI study demonstrated that stroke affected both the lesioned and contralesional hemispheres through functional connectivity analysis of fMRI findings (29). While another study found that although both static and dynamic functional network connectivity varied in patients with PSCI, only the mean dwell time (MDT) metric in dynamic functional network analysis correlated with patients’ MMSE scores.

Despite the variety of biomarker-based monitoring methods, EEG is becoming increasingly popular in the research community and clinical practice by virtue of its low cost, high usability, and ease of setup (30–32). Due to these advantages, EEG may have higher generalizability compared to fNIRS and fMRI, and has great potential for the universal identification of PSCI. Considering that there is currently no unfied criteria for classifying the stages of PSCI, this research attempts to divide PSCI into four stages from NC to server cognitive impairment, and tries to find the differences in EEG biomarker on these four levels of impairment, which on one hand, improves the framework of dividing the symptomatic stages of PSCI, and on the other hand, helps clinical workers to assess the stage of impairment of patients with PSCI, and facilitates individualized interventions and preventions for them.

## 2 Materials and methods

### 2.1 Participants information

This work recruited a diverse pool of participants, including both healthy controls and stroke patients exhibiting varying degrees of cognitive impairment. Specifically, a total of 32 participants were involved in the study, including 8 healthy control (*Mean* = 59, *SD* = 2.56), and 24 patients composing of 8 post-stroke patients with mild (*Mean* = 62, *SD* = 3.24), moderate (*Mean* = 61, *SD* = 3.57) and severe cognitive impairment individual (*Mean* = 65, *SD* = 1.85), respectively. This categorization enabled we to conduct a comprehensive analysis of the impact of stroke on cognitive abilities across varying degrees of severity. The research protocol strictly adhered to the ethical principles outlined in the Declaration of Helsinki, safeguarding the safety, rights, and dignity of all participants. The Institutional Review Board of the Research Center of Brain-Computer Interface at Chongqing Brain Cloud Sense Technology Co., LTD granted approval for the study, confirming its adherence to ethical standards and scientific validity. Prior to the commencement of the study, the experimenter provided detailed information to all participants regarding the objectives, procedures, and potential risks associated with the study. Ensuring informed consent, all participants signed a consent form, expressing their willingness to participate in the study.

### 2.2 Criteria for patient inclusion and clinical evaluation

The post-stroke group corresponding with the age range (50–80 years) was chosen for inclusion into the subject being evaluated. The individual exhibits moderate to severe paralysis, as assessed by a Brunnstrom score of IV or lower. Additionally, there are no other conditions that significantly affect the lower limbs. Finally, the individual does not suffer from any neurological or psychiatric disorders, and their medical condition is stable. Inclusion criteria for patients require them to be fully conscious, exhibiting symptoms or signs suggestive of cognitive impairment. Additionally, patients must have an NIHSS score ranging from 3 to 18 and a Montreal Cognitive Assessment (MoCA) score of ≤25, adjusted according to their level of education. At the time of randomised assignment, the patient must possess the ability to complete the Alzheimer’s Disease Assessment Scale (ADAS), cognitive subscale, extended version (ADAS-cog+) and the MoCA scale. Furthermore, all patients must provide written informed consent for their participation. Otherwise, the participants also fellow the standardized evaluated procedure in this work, and collected related results. This clinical evaluation process was conducted to determine the level of cognitive impairment among stroke patients in this work. The evaluation encompassed the following steps: (1) Collection of medical history: including stroke type, severity, location, prior neurological conditions, psychiatric illnesses, or trauma, as well as drug history, particularly the use of anticoagulants or antiplatelets; (2) Physical examination: focusing on a comprehensive neurological assessment to evaluate cognitive, motor, sensory, and coordination functions, and to identify any neurological localization signs, such as hemiplegia, aphasia, or hemianopia; (3) Cognitive screening using tools like the Simple Mental State Examination (MMSE) or Montreal Cognitive Assessment (MoCA) to detect cognitive impairment; (4) Neuroimaging: specifically cranial CT, to assess stroke severity and location, and rule out other potential causes of cognitive impairment; and (5) Diagnosis, where clinicians determined the severity of post-stroke cognitive impairment based on the findings from the medical history, physical examination, cognitive screening, and neuroimaging.

### 2.3 EEG data collection and preprocessing

In this study, closed-eye resting EEG data were collected from participants using a 44-channel electrode cap (NicoletOne EEG System, Natus Medical) with a sampling rate of 2 k Hz. The data were acquired according to the 10–20 International System, a standardized electrode placement method in EEG recording. Initially, the original 19 POL electrodes and 3 Cardinal electrodes were excluded due to missing specific locations, leaving 19 scalp electrodes for subsequent analysis. For data processing and analysis, MATLAB (R2021b, Mathworks, Natick, MA, United States) was used along with the EEGLAB toolbox (version 2023.1, Swartz Center for Computational Neuroscience, San Diego, CA, United States). The EEGLAB toolbox is a widely used and robust toolbox for EEG processing and analysis, providing a comprehensive set of tools for offline preprocessing of EEG data. Extensions for EEG data preprocessing, such as those developed by Delorme and Makeig (33), were also employed in this study. Data importation was facilitated by Biosig v3.8.1 (34), a software package that facilitates the importation and preprocessing of physiological signals. After importing the data, the localization of the channels was performed, and FCz was inserted as the reference electrode. Subsequently, the reference was re-set to A1 and A2 electrodes for better signal quality. Since the original sampling rate of the EEG data was 250 Hz, no resampling was necessary. However, to ensure accurate signal processing, a basic finite impulse response (FIR) filter was employed in this study. This filter is widely used in signal processing applications, as it offers good performance and stability.

Following the filtering step, the EEG data were low-pass filtered to remove line noise. The lower edge of the frequency pass band was set to 0.1 Hz to remove any unnecessary low-frequency components, while the higher edge was set to 120 Hz to dampen the low-frequency noise. This filtering step is crucial in EEG analysis, as it helps in removing artifacts and enhancing the signal-to-noise ratio. Next, the EEG data were decomposed using independent component analysis (ICA). ICA is a powerful statistical technique that allows the separation of independent sources in a multivariate dataset. In this study, the default Binica method in the EEGLAB toolbox was used for ICA decomposition. This method is known for its effectiveness in isolating and removing artifacts, such as eye movements or muscle activity, from the EEG signal. Finally, bad channels and bad EEG segments were removed using the EEGLAB raw program. This step is crucial to ensure data quality and reliability. By removing bad channels and segments, we can reduce the impact of artifacts and noise on subsequent analysis and interpretation of the EEG data. The rejected data were cleaned using the Clean Rawdata and ASR functions in EEGLAB, ensuring that only high-quality data were included in the final analysis.

### 2.4 Feature extraction in power spectral density

In this work, due to the small number of channels involved in the data, there is a lack of representativeness in demonstrating the individual brain functional areas. So, this work decided to use frequency domain analysis to extract the EEG features. PSD in stroke patients is an important feature for EG frequency domain analysis (35). In this study, using Fast Fourier Transform (FFT), each EEG signal was decomposed into five different frequency ranges: delta (0–4 Hz), theta (4–8 Hz), alpha (8–12 Hz), beta (12–30 Hz), and gamma (30–100 Hz). PSDs were computed using the Python toolbox (mne), and power averages were calculated for specific frequency ranges using the plot_psd_topomap function in the toolbox. In this way, features were constructed and topomap was plotted in each frequency band. Six contour lines were plotted in each brain topomap for comparative analysis.

### 2.5 Function connection map

We used python (version 3.8) to continue analyzing the data after preprocessing in EEGLAB. MNE-Connectivity (Larson et al. (2023). MNE-Python (v1.6.0). Zenodo)^1^ is an open-source Python package for connectivity. MNE-Connectivity is designed to be flexible and computationally efficient (36). This experiment uses the plot_connectivity_circle function in mne_connectivity.*viz.* for all-to-all connectivity estimation in the source space. The evaluation metric of connectivity in this experiment is the Phase-Lag Index (PLI), a measure that discards the phase distribution centered at 0 mod π to enhance robustness to the presence of a common source (37). The PLI is computed as Equation (1):


(1) $$PLI=\left|\langle \text{sign}\left(\Delta \varphi_{\text{rel}}\left(t\right)\right)\rangle \right|=\frac{1}{N}\sum_{n=1}^{N}\text{sign}\left(\Delta \varphi_{\text{rel}}\left(t_{n}\right)\right)$$

Where N denotes the time point, $\Delta \varphi_{\text{rel}}\left(t\right)$ denotes the phase difference between the two signals at time $t_{n}$, sign is a sign function whose output is 1 when the independent variable is positive, and −1 when the independent variable is negative. The PLI can take the value in the range of [0, 1], and the larger the value is, the stronger the phase synchronization between the two signals. The main advantage of PLI is its insensitivity to the volume conduction effect, but it seems to be more sensitive to noise.

### 2.6 Statistics processing

First, through analysis of averaged EEG data and brain topography maps in EEGLAB, we found that the MILD group experienced significant eye movement interference during data collection due to environmental factors. This led to inaccuracies in the biomarkers for the MILD group. To ensure the quality of subsequent comparative analyses, we have decided to exclude the MILD group from further analysis.

Then, we calculate the average power for each raw object in datalist as Formula (2). The formula for calculating power is shown below. Where x(n) is the signal of the brain wave on the frequency *n*.


(2) $$P\left(x\right)=\sum_{n}{\left|x\left(n\right)\right|}^{2}$$

Next, the subjects in each group were averaged to obtain the average value of 19 channels in each group, respectively. For a better understanding of the biomarkers of stroke, the EEG signal waves were divided into five bands. The standard is the same as when the PSD is decomposed. We set different thresholds to filter and group the previously obtained average signal values. The final output excel data contains four groups (nc, mci, mod., sev), each containing 19 channels and five different frequency bands. To further analyze the PSD differences in varies of cognitive impairment after stroke, one-way ANOVA and *post-hoc* permutation test with Bonferroni statistical methods were utilized to analyze the PSD values. In this study, the significance level is set to 0.001. And, the reported *p*-values were based on non-parametric permutation method.

## 3 Results

### 3.1 PSD results in different cognitive impairment groups

**Figure 1:**
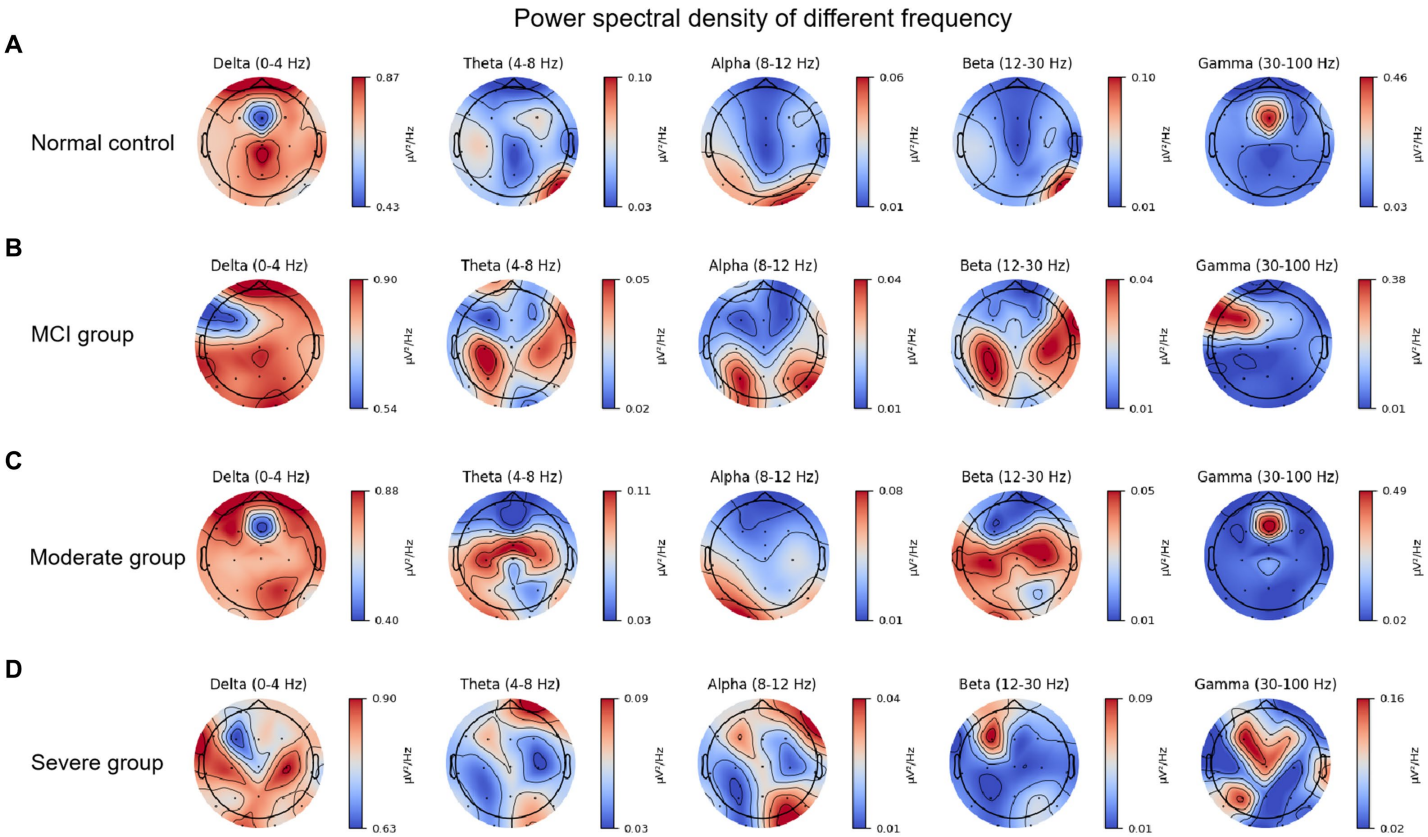
Power spectral density of different brain bands in various of patient groups: **(A)** Normal control. **(B)** Mild cognitive impairment. **(C)** Moderated cognitive impairment. **(D)** Severe cognitive impairment.

Figure 1 shows the PSD of 19 EEG channels in five bands for four groups composing of normal control, MCI, moderated and severe groups. To make this more concise, we use something like PSDnc to present power spectral density in certain band of normal control group. We elaborate on our observation results by frequency band. Specifically, in the delta band, all groups in the frontal area are significantly superior PSD value compared to the other frequency bands. Despite PSDsevere, the other groups showed relatively strong PSD values in the frontal pole area. Interestingly, PSDnc and PSDmci both show high value in central zero area, but the priority disappears in PSDsevere. And, PSDsevere fluctuated in [0.63, 0.90], which has a higher value of lower boundary than other groups. PSDmci shows a lower upper bounds of 0.05 than other groups in the theta band. And, we can notice that both PSDmci and PSDmod show high value in bilateral central-parietal area, while PSDnc and PSDsevere not. PSDsevere shows relative high value in right oppicital/right frontal pole. In the alpha band, the condition is similar to the theta band, but we can find that the overall PSD value have decreased. PSDmod and PSDsevere decrease most obviously with the range of [0.01, 0.08] and [0.01, 0.04], respectively. What is noteworthy is that high value in bilateral central-parietal area disappears of PSDmod. In the beta band, high value in bilateral central-parietal area has a tendency to expand in PSDmci group. And, PSDmod in bilateral central-temporal area has reappeared with relatively high values. More noticable is that PSDsevere’s boundary value increases significantly, which is ranged in [0.01, 0.09], and only the frontocentral area of the whole brain showed a strong PSD value. PSD in gamma band shows an significant difference. The different group’s range gap increases, upper boundary is apparently higher than theta, alpha and beta band, just below the delta band. By comparing the boundary values, we find that the strength of PSDsevere is significantly low, which fluctuated in [0.02, 0.16]. And, the PSDsevere was more dispersed at the frontal center area compared with the other groups.

**Figure 2:**
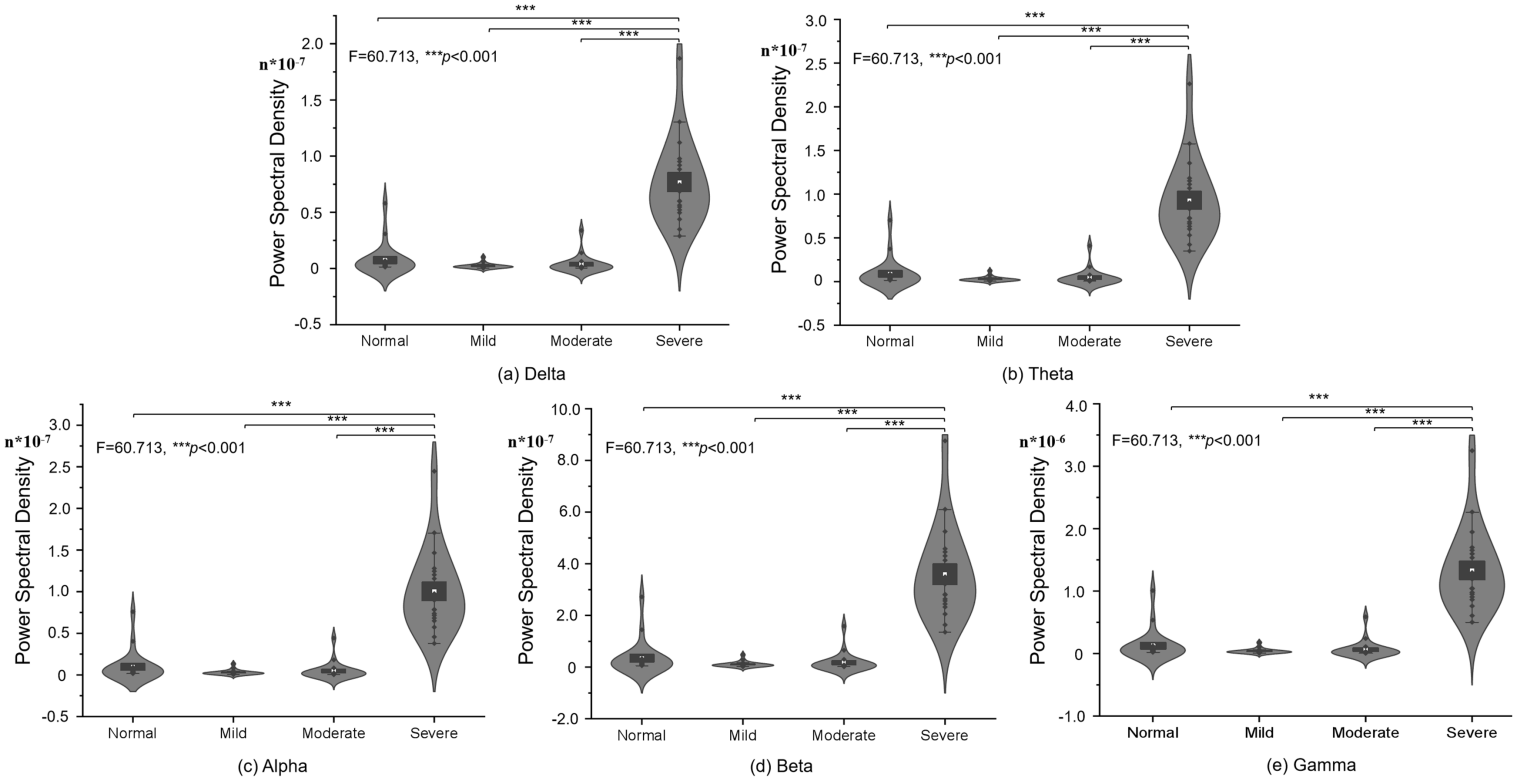
PSD statistical difference of different frequency in different cognitive impairment groups: **(A)** Delta. **(B)** Theta. **(C)** Alpha. **(D)** Beta. **(E)** Gamma frequency bands.

For further quantification of the PSD results, one-way ANOVA and *post hoc* methods were used to analyze the PSD of different groups and present the visualization results in the Figure 2. One-way ANOVA was chosen due to its applicability in comparing means across multiple groups simultaneously. *Post hoc* tests help identify specific group differences while controlling for Type I errors that may arise from multiple comparisons. And, the Figure 2 showed the PSD differences of various of cognitive impairment at different brain brand frequency, which is composing of delta, theta, alpha, beta and gamma frequency. Figure 2A showed the PSD difference of normal control, mild, moderated and severe cognitive impairment groups at delta brain band, and the cerebral PSD was significant difference between severe cognitive impairment group and normal control, mild, moderate groups (*F* = 60.713, *p* < 0.001); Similarity, Figures 2B–D showed the PSD difference of normal control, mild, moderated and severe cognitive impairment groups at theta, alpha, beta and gamma brain bands, respectively. Specifically, all statistical visualized results show the cerebral PSD significant difference between severe cognitive impairment group and normal control (*F* = 60.713, *p* < 0.001), mild (*F* = 60.713, *p* < 0.001), moderate groups (*F* = 60.713, *p* < 0.001). Detailed statistical results were shown in the Table 1, and the gamma frequency of severe cognitive impairment group (Mean = 1.335e-6, SE = 6.53e-7) was most significant difference compared to the mild cognitive impairment group (Mean = 4.01e-8, SE = 4.02e-8), and superior significant difference was shown between servere cognitive impairment groups and normal control (Mean = 1.293e-7, SE = 2.416e-7), moderate cognitive impairment group (Mean = 6.42e-8, SE = 1.342e-7) at the gamma frequency bands.

**Table 1:** Detailed statistical results of different cognitive impairment groups.

Brain band	Group name	Mean	Std. E	*F*	*p*
Delta	MCI	2.30e-9	2.30e-9	60.713	0.000***
	Mod	3.70e-9	7.70e-9		
	NC	7.40e-9	1.39e-8		
	Sev	7.68e-8	3.76e-8		
Theta	MCI	2.80e-9	2.80e-9	60.713	0.000***
	Mod	4.50e-9	9.30e-9		
	NC	9.00e-9	1.68e-8		
	Sev	9.30e-8	4.55e-8		
Alpha	MCI	3.00e-9	3.00e-9	60.713	0.000***
	Mod	4.80e-9	1.01e-8		
	NC	9.70e-9	1.82e-8		
	Sev	1.005e-7	4.92e-8		
Beta	MCI	1.08e-8	1.08e-8	60.713	0.000***
	Mod	1.73e-8	3.62e-8		
	NC	3.48e-8	6.51e-8		
	Sev	3.598e-7	1.76e-7		
Gamma	MCI	4.01e-8	4.02e-8	60.713	0.000***
	Mod	6.42e-8	1.342e-7		
	NC	1.293e-7	2.416e-7		
	Sev	1.335e-6	6.53e-7		

### 3.2 Functional connection map for different cognitive impairment

**Figure 3:**
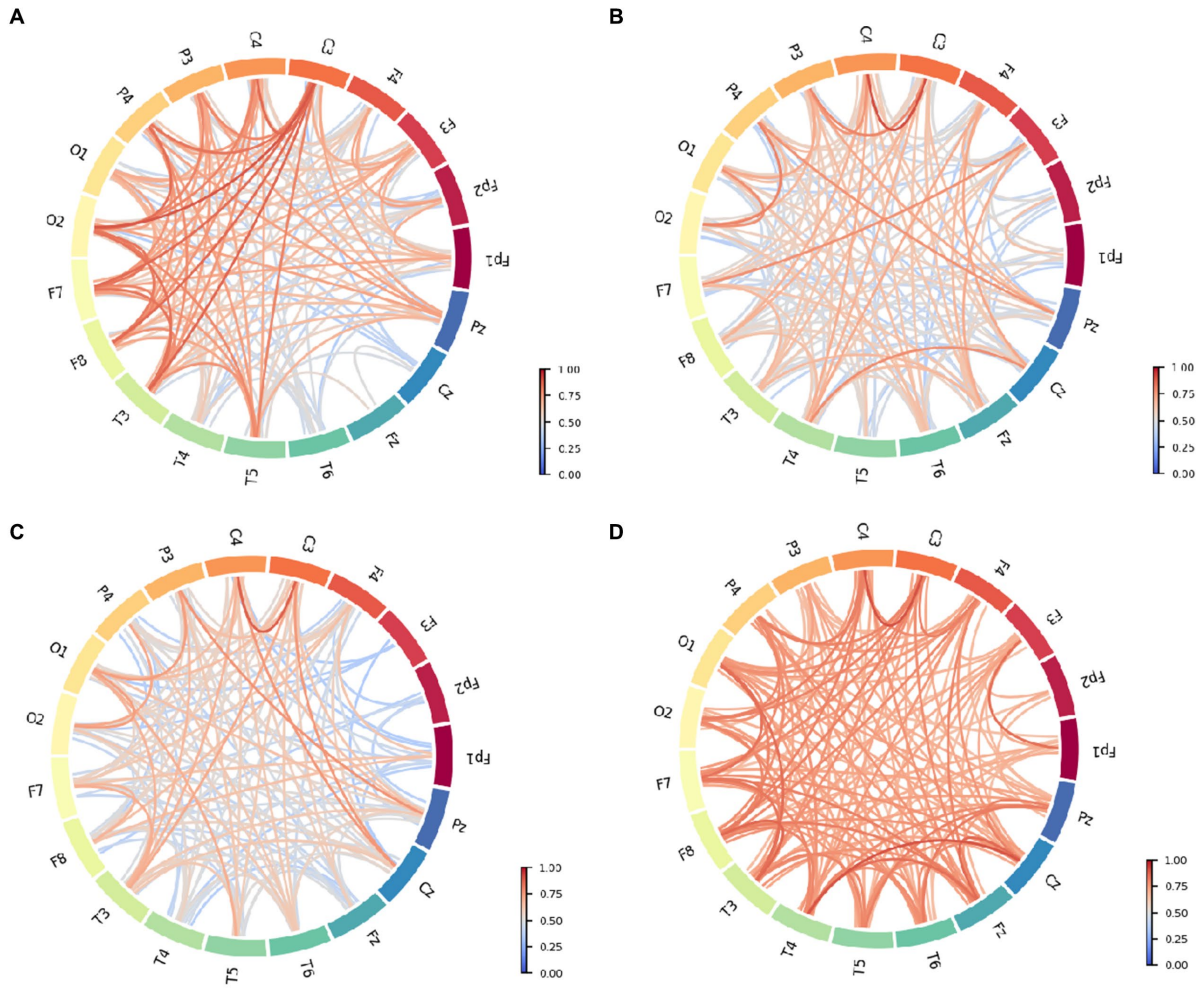
Functional connective circle map of different patient groups: **(A)** Normal control. **(B)** Mild cognitive impairment. **(C)** Moderate cognitive impairment. **(D)** Severe cognitive impairment.

The functional connectivity of each group is shown in Figure 3. Intuitively, the severe group appears to have stronger functional connectivity compared to the other groups, and the moderate group has overall weaker relative connectivity. The strength of the PLI that showed stronger connectivity was mainly related to left–right interhemispheric connections, anterior temporal (e.g., F7, F8), occipital (e.g., O1, O2) and mid-temporal (e.g., T3, T4) connections, central (e.g., C3, C4), and details can be seen in Figure 3A. Observing Figure 3B compared to functional connection of normal control (FCnc), FC_mci interhemispheric temporal (e.g., T3, T4, T5, T6) connections were significantly weakened. Bilateral frontocentral-parietal connections (e.g., C3, C4, PZ, P3, P4) connections are largely maintained. And, connections in frontocentral-central (e.g., Fz, Cz, C3, C4, C6) areas exhibit stronger PLI values. Interestingly, connections in frontocentral-central (e.g., Fz, Cz, C3, C4, C6) areas exhibit stronger PLI values. The overall PLI strength of FCmod (see Figure 3C) is weaker than that of FCmci, which is evident mainly in the middle line channels (e.g., FCz, Cz, Pz) and right-temporal (e.g., T4, T6) areas. Bilateral frontocentral-parietal (e.g., F3, F7, C3, P3; F4, F8, C4, P4) connections are weakened. In addition to the high PLI value in the region with high strength of FCsevere connection, it shows higher PLI strength between middle line channels (e.g., FCz, Cz, Pz), which is shown in Figure 3D. And the middle line channels also show stronger connections with the occipital, temporal, and parietal. At the same time, the connection between the left and right hemispheres is also significantly enhanced (e.g., O1, O2, T3, T4, T5, T6, F3, F4). These results suggest that different cognitive state groups have distinct connectivity preferences that correspond to unique patterns of functional connectivity. And, the overall core structure or underlying framework of connectivity remained relatively stable despite pathological states or other changes. The central (e.g., C3, C4,Cz) area has remained strongly connected. The internal connectivity of the oppicital and parietal area has been less affected, and has maintained a certain PLI value. For FCnc, FCmod and FCmci, their connectivity are weakened to different degrees, mainly in frontal and temporal area, but the main frame is preserved. On the other hand, FC severe shows the enhancement of the overall connectivity on the basis, especially the middle line channels.

## 4 Discussion

The findings of the study indicated the existence of notably elevated brain activity within the Delta frequency range, indicating the Delta frequency range’s crucial involvement in certain brain functions. Additionally, among the cohort of patients experiencing severe cognitive impairment, the Delta frequency range exhibited augmented neural activity confusion and uneven cortical power distribution. This observation implies that neural activity within the Delta frequency range may undergo alterations in the brains of patients with severe cognitive impairment, leading to an unbalanced power distribution (38). The processing capacity exhibits a distinct pattern, possibly indicating significant dysfunction within specific brain regions. Patients diagnosed with mild cognitive impairment (MCI) displayed a notably elevated cortical power concentration in the left prefrontal region. This augmented neural activity might elevate the risk of concussion, thereby influencing cognitive performance (39). Such concussion could be indicative of cognitive shifts, potentially signaling a more severe cognitive decline, akin to Alzheimer’s disease (40). Among those with severe cognitive impairment, there was an observed elevation in neural activity and cortical power in the right prefrontal region, particularly within the theta and alpha bands. Similarly, patients with MCI demonstrated comparable patterns of cortical activity, concussion trends, and perturbations across the theta, alpha, and beta bands. These observations could suggest an early stage of brain function deterioration (41). However, at this stage, brain activity remained relatively stable. Together, these findings offer unique insights into the brain activity patterns of individuals with cognitive impairment, providing valuable clues for further research and informing the development of novel therapeutic strategies. Furthermore, patients with moderate cognitive impairment exhibited notable alterations in the cortical power signal. Specifically, there was a significant increase in activity intensity within the theta and beta frequency bands in the occipital and parietal regions. These changes suggest a more disordered neural activity in these brain regions, potentially affecting information processing and cognitive functions. A comprehensive understanding of cognitive impairment disparities post-stroke is essential. To this end, the present study conducts a thorough examination and meticulous analysis of functional connectivity among various brain regions across distinct patient cohorts. Notably, individuals experiencing severe cognitive impairment exhibit a high degree of connectivity within neural pathways across different brain regions, maintaining remarkable consistency in connection strength. This observation suggests that the coordinated interaction among brain regions in the context of severe cognitive impairment may underlie compensatory neural activity, consistent with research postulates by researchers (42). Conversely, among patients who maintain normal cognitive function after stroke, the functional connectivity within the brain area exhibits a leftward bias, with higher connectivity strength in the left hemisphere.

The potential association between the functional reorganization of the brain post-stroke and the subsequent reduction in the functionality of the right brain region, coupled with a relative augmentation in the left brain region, has been explored by Grefkes and Fink (43). Notably, this lateralization pattern was not observed among patients with mild to moderate cognitive impairment, suggesting that the functional connectivity within brain regions remains relatively intact in cases of less severe cognitive impairment following stroke. These observations hold significant value in enhancing our understanding of the neural mechanisms underlying cognitive dysfunction after stroke and offer novel insights and strategies for clinical interventions in the future. Furthermore, this study identified distinct variations in functional connectivity among different patient cohorts. This suggests that the severity of cognitive impairment after stroke is not random or accidental, but is closely related to the functional connectivity status of the brain. Specifically, the following crucial correlation are declared in the previous researches: (1) Reduced functional connectivity in affected bran regions: Impaired memory was linked to reduced connectivity within the hippocampus and other medial temporal lobe structures (44, 45); (2) Impaired inter-hemispheric connectivity: Stroke often affects one side of the brain, leading to disruption of inter-hemispheric connectivity between the affected and unaffected hemispheres. This impaired connectivity can contribute to cognitive deficits, as many cognitive processes require the coordinated activity of both hemispheres (29, 46); (3) Network-level alterations: disrupted connectivity within the default mode network (DMN) has been linked to impaired self-referential processing and memory retrieval (29); (4) Correlation with cognitive performance: a significant correlation between the severity of cognitive impairment and the degree of functional connectivity disruption in specific brain networks. For example, reduced connectivity within the frontoparietal network has been associated with impaired executive function, while decreased connectivity in the language network has been linked to language deficits (29, 44); (5) Predictive value: Changes in functional connectivity can predict cognitive outcomes after stroke. For example, stroke survivors with greater improvement in functional connectivity over time have been found to experience better cognitive recovery (45, 46).

Certainly, it is crucial to acknowledge the inherent constraints within this research. Firstly, the scale of the sample used was relatively limited, potentially limiting its applicability to a wider population. A smaller sample size may have introduced biases or led to inconclusive results, thus posing challenges in drawing definitive conclusions regarding the mechanisms of cognitive impairment post-stroke. Secondly, the study’s design was retrospective, meaning the data were collected and analyzed retrospectively. While this approach is useful in identifying patterns or trends, it cannot provide causal evidence or predictive insights into future outcomes. To gain a thorough understanding of the longitudinal impact of stroke on cognitive function, a prospective long-term follow-up study is imperative. Such a study would enable researchers to observe participants over time, capturing the natural progression of cognitive impairment and its relationship to EEG biomarkers. However, despite its limitations, this study explores novel approaches to investigate the mechanisms of post-stroke cognitive impairment using EEG techniques, laying a foundation for further intensive research. This study presents a novel approach to examining the neural mechanisms of cognitive impairment following stroke. While some limitations exist, this study offers a valuable tool for the early detection and diagnosis of cognitive impairment, serving as a reference for interventions aimed at improving cognitive function.

## 5 Conclusion

This research has identified that EEG biomarkers effectively discriminate different degrees of cognitive decline among stroke sufferers. Our findings establish a strong link between the extent of cognitive impairment post-stroke and the functional connectivity of the brain. These revelations offer a fresh perspective for the early detection and diagnosis of cognitive decline after stroke, paving the way for the development of innovative therapeutic strategies. Furthermore, these revelations deepen our understanding of the intricate relationship between the brain’s functional connectivity and cognitive impairment. By thoroughly examining this relationship, we can gain further insights into the development of novel treatment options. This may encompass mitigating cognitive impairment symptoms by optimizing the brain’s functional connectivity or enhancing cognitive function through modulation of neural activity. In conclusion, this study presents novel ideas and approaches for the diagnosis and treatment of cognitive impairment in stroke patients. By leveraging EEG biomarkers and information regarding the brain’s functional connectivity, we can enhance the precision of cognitive impairment diagnosis and treatment, thereby enhancing the overall quality of life for stroke patients.

## Data availability statement

The raw data supporting the conclusions of this article will be made available by the authors, without undue reservation.

## Ethics statement

This work was conducted based on the tenets of the Declaration of Helsinki, and the protocol was approved by the Institutional Review Board of the Research Center for Brain-computer Interface, Chongqing Brain Cloud Sense Technology Co., LTD. Finally, The experimenter informed the participants of the detailed process and risks, and all participants were signed the informed consent before this study.

## Author contributions

MX: Investigation, Validation, Visualization, Writing – original draft. YucZ: Methodology, Validation, Visualization, Writing – original draft. YueZ: Investigation, Validation, Writing – original draft. XL: Supervision, Validation, Writing – review & editing. KQ: Resources, Supervision, Validation, Visualization, Writing – original draft, Writing – review & editing.

## Footnotes

^1^ https://doi.org/10.5281/zenodo.10161630
